# Chitosan-Grafted Carbon Oxynitride Nanoparticles: Investigation of Photocatalytic Degradation and Antibacterial Activity

**DOI:** 10.3390/polym15071688

**Published:** 2023-03-28

**Authors:** Xuemei Bai, Jingmin Luan, Tingting Song, Haifeng Sun, Yuhua Dai, Jianxiang Yu, Huafeng Tian

**Affiliations:** 1 School of New Materials and Chemical Engineering, Beijing Institute of Petrochemical Technology, Beijing 102617, China; 2 College of Chemistry and Materials Engineering, Beijing Technology and Business University, Beijing 100048, China

**Keywords:** carbon oxynitride, CS grafting, MB degradation, antibacterial properties, photocatalytic activity

## Abstract

In this work, a series of chitosan (CS)-grafted carbon oxynitride (OCN) nanoparticles (denoted as CS-OCN) were successfully synthesized for the first time by thermal polycondensation and subsequent esterification. The structure and photocatalytic performance of CS-OCN nanoparticles were investigated. The XPS spectra of CS-OCN-3 showed the presence of amino bonds. The optimal photocatalytic degradation efficiency of the synthesized CS-OCN-3 could reach 94.3% within 390 min, while the photocurrent response intensity was about 150% more than that of pure OCN. The improved photocatalytic performance may be mainly attributed to the enhanced photogenerated carrier’s separation and transportation and stronger visible light response after CS grafting. In addition, the inhibition diameter of CS-OCN-3 reached 23 mm against *E. coli* within 24 h under visible light irradiation, exhibiting excellent photocatalytic bactericidal ability. The results of bacterial inhibition were supported by absorbance measurements (OD600) studies of *E. coli*. In a word, this work provided a rational design of an efficient novel metal-free photocatalyst to remove bacterial contamination and accelerate the degradation of organic dyes.

## 1. Introduction

In recent decades, the problem of environmental pollution has been becoming increasingly serious, especially in the field of water pollution [1,2,3]. The growing levels of bacteria and organic pollutants in wastewater are severe problems to be addressed. The organic pollutants and bacteria can be eliminated by some reliable techniques, including physical adsorption [4,5], biological treatment [6] and chemical flocculation [7]. Unfortunately, these traditional methods are not efficient and prone to generate pollutants secondarily [8].

Photocatalytic degradation is considered as one of the ideal methods for the removal of dyes and other organic chemicals from water due to its high photocatalytic activity and the efficient use of solar energy [9]. According to photodegradation mechanism, organic dye molecules are photochemically broken down into simpler, lower molecular weight and non-toxic components with the aid of photocatalysts [10]. Semiconductor photocatalysts can be activated by absorbing light, which will accelerate photodegradation reactions without consumption [11]. Photocatalysis has emerged as a potential technology for the complete removal of most harmful chemicals and bacteria from wastewater with the wide application of metal oxide nanoparticles [12,13]. For example, zinc oxide (ZnO) [14,15], bismuth oxide (Bi_2_O_3_) [16] and titanium dioxide (TiO_2_) [17,18] have been used as photocatalysts and antimicrobial agents. However, these metal oxides can only be activated by high-energy photons (λ < 400 nm) and hence with very low solar efficiency (4–5%) [19]. In addition, the diffusion of metal ions also causes secondary pollution of water resources. In contrast, graphitic carbon nitride (g-C_3_N_4_), which belongs to a two-dimensional material, has attracted much attention for its potential applications in the degradation of organic pollutants [20], water splitting [21], CO_2_ fixation [22,23] and biological applications [24]. The g-C_3_N_4_ is composed of carbon and nitrogen elements and synthesized by thermal polycondensation of precursors such as melamine, urea, cyanamide and dicyandiamide [25,26,27,28], which is facile to be produced on an industrial scale. Possessing a suitable energy band structure (2.7 eV), g-C_3_N_4_ is favorable for visible light absorption (460 nm), and it facilitates simple band gap design, modification and doping [29,30,31]. Liu et al. [32] reported that the photocatalytic degradation efficiency of the prepared AgCl/CNTs/g-C_3_N_4_ was 86.44%, which was three times higher than that of pure CN, and the composite showed a strong inhibitory effect on *E. coli*. The novel heterojunction pCN/TNs composite exhibited excellent photocatalytic performance (86.2% degradation rate) for the degradation of tetracycline (TC) under visible light irradiation for 120 min [33].

However, the practical application of g-C_3_N_4_ is still hindered by the agglomeration of nanoparticles and low photocatalytic efficiency. It was found that combining photocatalysts with polymers increased the surface area of the catalytic system, thus improving the hydrolytic stability and chemical stability of the photocatalysts [34]. Qi et al. [35] prepared g-C_3_N_4_-based aerogels with different ratios of carboxymethyl cellulose and β-cyclodextrin, and they achieved a 97.99% removal of RhB within 90 min. Xing et al. [36] reported that the prepared PEI/OCN had excellent rapid photocatalytic bactericidal ability, with an inactivation efficiency of 7.0 log CFU/mL against *E. coli* under visible light irradiation for 30 min. Hou et al. [37] prepared PANI/g-C_3_N_4_ composites with good photoanodic antifouling/antibacterial properties under visible light irradiation (96.5% and 95.3% killing of *E. coli* and *S. aureus*, respectively, after 30 min of visible light irradiation). Sun et al. [38] reported that novel highly efficient composite PoPD/AgCl/g-C_3_N_4_ nanosheets with large specific surface areas had good degradation efficiency for tetracycline. Nevertheless, many composite photocatalysts also have some shortcomings, such as low structural stability and recycling stability. Therefore, the construction of structurally stable, recyclable and efficient composite photocatalysts for industrial practical applications remains a great challenge.

CS is a natural cationic polymer with highly reactive amino and hydroxyl groups, which could be easily covalently bound with the photocatalysts, and it has a stable crystal structure accounting for insoluble in water, alkali and most organic solvents [39]. It was reported that CS and oxidized metal photocatalytic systems have enhanced light irradiation area and improved synergistic photocatalysis [34]. In a previously reported study, Yang et al. [40] prepared an easily regenerated TiO_2_-impregnated CS adsorbent to achieve the adsorption and degradation of pollutants. In addition, CS itself possesses antibacterial properties, and the above properties of CS could be utilized to combine it with g-C_3_N_4_ in the form of covalent bonds, which is expected to generate composite catalysts with better photocatalytic and antibacterial abilities.

**Scheme 1:**
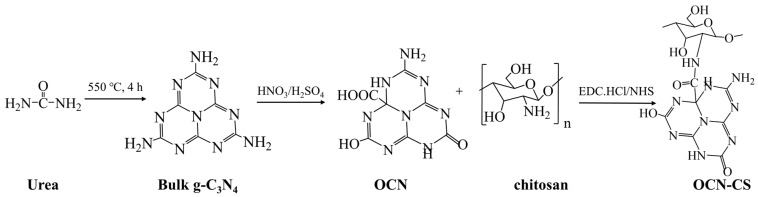
Schematic diagram of the preparation of CS-grafted OCN nanoparticles.

Herein, the natural cationic polymer CS was successfully grafted onto the photocatalytic OCN nanoparticles for the first time by thermal polycondensation and subsequent esterification with covalent bonding. We investigated the relationship between the photocatalytic activity and the graft ratio of obtained CS-OCN, and we deeply researched the bacteria inhibition property and its crucial factors. The obtained nanoparticles were characterized to study their physical properties, including morphological, structural and optical properties. The degradation performance of methylene blue (MB) dye was to evaluate its photocatalytic performance, and its antibacterial ability against *E. coli* was measured by the inhibition circle method. The results show that an optimal ratio exists between OCN and CS, which is promising to obtain the best photocatalytic and antibacterial activities. So, a reliable technical way is provided to prepare an environmentally friendly photocatalyst. The specific preparation process is described in Scheme 1.

## 2. Materials and Methods

### 2.1. Materials

Urea (99.9%) was purchased from Macklin (Shanghai, China). Chitosan powder (Mw = 300,000) was supplied from Zhejiang Golden Shell Biopharmaceutical (Yuhuan, China). Acetic acid (99.7%), nitric acid (69%), sulfuric acid (98%), hydrogen peroxide (30%) were analytical reagents and were purchased from Beijing Chemical Works (Beijing, China). *N*-hydroxysuccinimide (NHS), 1-(3-Dimethylaminopropyl)-3-ethyl carbodiimide hydrochloride (EDC.HCl, 98%) were of analytical grade and were obtained from Aladdin Biochemical Technology (Beijing, China).

### 2.2. Fabrication of CS-Grafted OCN

OCN nanosheets were synthesized with reference to the previously reported method [41]. The g-C_3_N_4_ was prepared by thermal polymerization of urea with a heating rate of 10 °C/min at 550 °C for 4 h. Then, the g-C_3_N_4_ powder was added to 20 mL (1:1) of a mixture of concentrated H_2_SO_4_ and HNO_3_, heated at 45 °C for 2 h by ultrasonication, and then H_2_O_2_ was added and further sonicated for 2 h to obtain white OCN. The OCN (0.025, 0.05, 0.1, 0.2, 0.6 g) was added to 100 mL of ultrapure water and sonicated for 30 min to form uniform OCN dispersions. Then, 0.3 g of EDC.HCl and 0.15 g of NHS were added to the dispersion and stirred magnetically at room temperature for 12 h. Then, 0.2 g of CS (dissolved in 20 mL of 2% acetic acid solution) was added and stirred vigorously at room temperature for another 12 h (the mass ratios of CS: OCN were 8:1, 4:1, 2:1, 1:1 and 1:3, respectively). After that, the CS-OCN reaction solution was vacuum filtered three times with a 0.2 µm filter membrane. Finally, the CS-grafted OCN powder, denoted as CS-OCN-1, CS-OCN-2, CS-OCN-3, CS-OCN-4, and CS-OCN-5, was obtained by vacuum drying at 60 °C.

### 2.3. Characterization

The crystal structures of OCN and CS-OCN were investigated by X-ray diffraction analysis (Japan-Riken-Ultima IV) in the 2*θ* range (10–100°) with Cu K*α* (*λ* = 1.54 Å) radiation. The functional groups were identified using FTIR-850 in the range of 4000–600 cm^−1^. The transmission electron microscopy (TEM) images were obtained using a USA-FEI-Tecnai G2 F20 at an accelerating voltage of 200 kV. TGA measurements (Setline TGA) were carried out under N_2_ gas environment at a heating rate of 10 °C/min from 25 to 800 °C. The elemental composition of OCN, CS-OCN and its chemical state were analyzed by X-ray photoelectron spectroscopy (XPS) (Thermo Scientific Escalab 250Xi, Thermo Fisher, Waltham, MA, USA). The UV absorption spectra of OCN, CS-OCN were recorded in the range of 220–800 nm using a UV-3600 UV spectrophotometer. The PL spectra were recorded with an Edinburgh FLS 980 spectrometer (350 nm excitation). The measurements of the transient photocurrent response (0.5 V, 300 W), and electrochemical impedance spectra of the photocatalysts were conducted in the three-electrode system of CHI660E electrochemical workstation(Chenhua, Shanghai, China), where Pt was used as the counter electrode, Ag/AgCl as the reference electrode, the prepared samples were coated into a thin film (1 cm^2^) on FTO glass as the working electrode, and the electrolyte solution was 0.5 mol/L Na_2_SO_4_ solution.

### 2.4. Reactive Oxygen Species (ROS) Measurements

ROS generation of OCN and CS-OCN-3 was measured with 2-(3,6-diacetoxy-2,7-dichloro-9H-xanthene-9-yl) benzoic acid (H2DCFDA) as indicator. Briefly, 1 mL of 0.5 mg/mL aqueous H2DCFDA solution was added to 3.2 mL of 0.01 M aqueous KOH solution. The solution was incubated for 30 min at 30 °C and neutralized with 17.2 mL of PBS solution to obtain a concentration of 0.024 mg/mL of H2DCFDA solution. OCN or CS-OCN-3 (0.5 mg/mL, 50 μL) was mixed with 1.95 mL H2DCFDA solution and irradiated with an Xe lamp (20 mW/cm^2^) for 0, 2, 4, 6, 8 and 10 min. After that, absorption spectra and fluorescence intensity were measured with Synergy HTX.

### 2.5. Antibacterial Activity

The inhibitory activity of OCN, CS-OCN against *E. coli* (ATCC 8739) was evaluated by the zone of inhibition method [42]. First, 100 µL of 10^7^ CFU/mL bacterial suspension was spread on LB agar plates, and then UV-sterilized OCN, CS-OCN nanoparticles with a diameter of 6 mm were placed on the agar surface. After irradiation with a 300 W xenon lamp for 10 min and then incubation in a 37 °C bacterial incubator for 24 h under D50 fluorescent lamp (400–780 nm), the diameter of the inhibition zone was measured. The inhibition zone was recorded based on the average diameter of the transparent area. To further quantified the inhibition performance, optical density absorption value (OD600) measurements were performed. The concentration of the above bacterial suspension (10^7^ CFU/mL) was adjusted to OD 600 nm = 0.1. Equal amounts of OCN, CS-OCN-1, CS-OCN-2, CS-OCN-3, CS-OCN-4 and CS-OCN-5 (90 µg/mL) were mixed with bacterial suspension (10 mL) and exposed to a 300 W xenon lamp for 10 min. The effect of UV light on *E. coli* in this study could be ignored because the small portion of UV light was like natural sunlight. Bacterial suspensions without any treatment were taken as control group. Each sample was incubated at 37 °C for 24 h. The change in OD600 was measured at certain time intervals and the inhibition curve was plotted. The experiments for antibacterial performance were performed in triplicate.

### 2.6. Photocatalytic Performance Assays

A 500 W xenon lamp was used to simulate sunlight (λ > 420 nm), the photocatalytic performance of different samples was investigated by degrading methylene blue (MB) at room temperature. The specific operation steps were as follows: The 40 mg/L solution of MB (30 mL, 30 mg of photocatalyst) and 10 mg/L solution of MB (30 mL, 10 mg of photocatalyst) were placed in a beaker. Before turning on the light, continuous stirring was performed at a dark and constant temperature for 30 min in order to reach the adsorption–desorption equilibrium. After that, the xenon lamp was turned on and samples were taken at intervals and centrifuged at 8000 rpm. Then, 4 mL of the supernatant was taken into a quartz cuvette and the absorbance was measured using a UV-Vis spectrophotometer. The absorbance value at the maximum absorption wavelength of 664 nm was recorded. The degradation efficiency of the samples to the MB solution was calculated according to Equation (1).

(1) $$\eta =\frac{C_{0}-C_{t}}{C_{0}}\times 100\%$$

where η is the rate of degradation, and $C_{0}$ and $C_{t}$ are the concentrations of MB solutions at the end of the dark reaction and at different reaction moments, respectively.

## 3. Results and Discussion

### 3.1. Structure and Composition of Prepared CS-OCN

**Figure 1:**
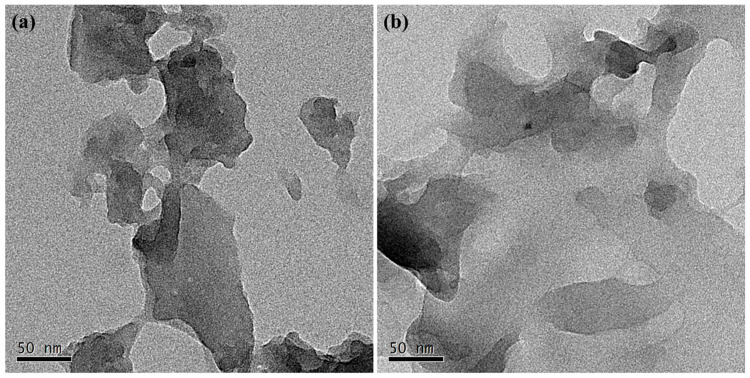
TEM images of (**a**) OCN and (**b**) CS-OCN-3.

The morphology of the OCN and CS-OCN were observed by TEM (Figure 1) and SEM (Figure S1). The TEM images of the OCN (Figure 1a) and CS-OCN-3 (Figure 1b) both showed the nanosheet layers and porous structures. CS-OCN-3 displayed a thinner lamellar structure with fewer layers, while OCN was dense. The ultra-thin lamellar and porous structure was very favorable to exposing more catalytic active sites [31], and it was more reactive to visible light, improved light absorption and enhanced molecular diffusion kinetics, which led to improved photocatalytic performance [43]. The OCN and CS-OCN nanoparticles were sonicated and dispersed in PBS solution to obtain a 1 mg/mL suspension to investigate their stability during storage. As shown in Figure S2, the suspensions were still uniformly dispersed after 3 days, which indicated that the OCN and CS-OCN nanoparticles had good stability during storage.

**Figure 2:**
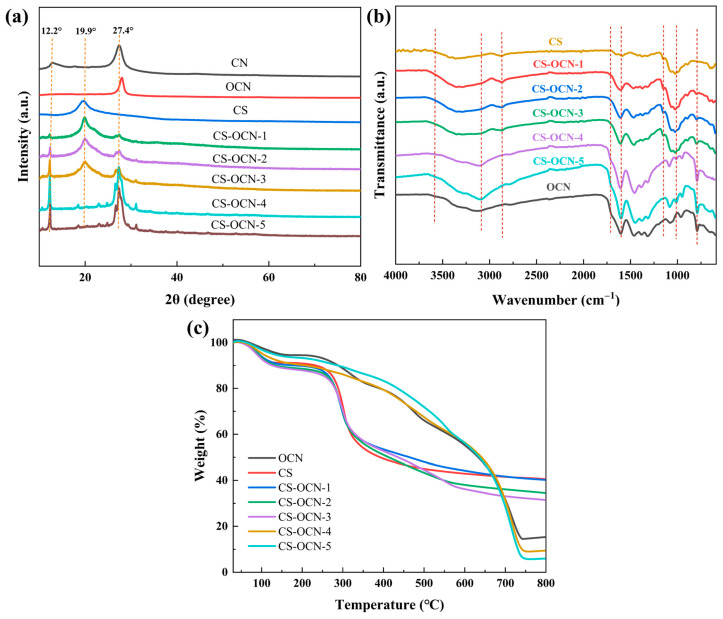
(**a**) XRD patterns, (**b**) FT-IR spectra and (**c**) TGA curves of OCN and CS-OCN.

The crystal structures of the prepared samples were analyzed by X-ray diffraction mapping (XRD). As shown in Figure 2a, the peak at 12.2° corresponded to the (100) crystal plane of OCN, while 27.4° belonged to the (002) crystal plane of OCN [44]. The two diffraction peaks were mainly attributed to the stacking of repeating units and interlayer structures within the OCN surface. Apparently, CS-OCN had three peaks at 12.2°, 19.9° and 27.4°, respectively, which indicated that the crystal structures of OCN and CS were not changed [45,46], and among them, the intensity of the peaks at 12.2° and 27.4° increased with the ratio increase in OCN. The diffraction peak at 19.9° nearly disappeared in CS-OCN-4 and CS-OCN-5, probably because the amount of CS grafting became less and the CS grafted uniformly on the OCN. The crystallinity index (CrI) values for all peaks are summarized in Table S1.

FT-IR (Figure 2b) further revealed the molecular structure changes in the prepared samples. It can be found that the FT-IR spectrum of CS-OCN was basically the same as that of OCN, but with slight changes in some regions. It indicated that the primary structure of OCN was well preserved after grafting. Among them, the band between 3000 and 3600 cm^−1^ was caused by the stretching vibrations of residual N-H bonds, O-H bonds and adsorbed H_2_O [47]. Typical tensile and bending vibrations of the tri-s-triazine ring appeared in the vibrational band in the range of 1100–1650 cm^−1^ [48]. The peaks at 2870 cm^−1^ were related to CH and CH_2_ vibrations, and the intensity of this peak decreased with the lower CS content. Additionally, the sharp peak at 802 cm^−1^ was assigned to the respiratory mode of the tri-s-triazine ring [49]. The intensity of this peak increased with increasing OCN content, indicating that more tri-S-triazine rings were introduced. The enhanced intensity of the C=O peak at 1712 cm^−1^ and the N-H vibrational peak at 1606 cm^−1^ may be due to the generation of an amide bond (-CO-NH-) between CS and OCN. This result was further demonstrated by XPS analysis.

Thermal stability was studied by thermogravimetric analysis (TGA). The TGA and DTG curves of OCN and CS-OCN are shown in Figure 2c and Figure S3. The first weight loss of CS-OCN and OCN was below 200 °C, probably due to the adsorption of water. The higher the amount of CS, the more weight was lost, up to about 12.17%, while the OCN weight loss rate was only 6.67%. In the temperature range of 200–360 °C, the second weight loss of OCN was 13.91%, which could be attributed to the decomposition of oxygen-containing functional groups introduced by the oxidation process. As for CS-OCN, the decomposition of CS began at 260 °C. It was probably the rapid destruction of the O-H and C-O bonds in the CS molecule, accompanied by the loss of a large amount of hydrogen, oxygen and a small amount of carbon. When the temperature was higher than 500 °C, the third weight loss occurred, and the tri-s-triazine structural unit of OCN started to decompose [50]. The CS-OCN-1,2,3 had similar weight loss curves to and higher residues than CS, probably due to high CS grafting. CS was carbonized at temperatures above 500 °C. The residue was the result of carbonization. However, with the decrease in CS grafting, the weight loss curves of CS-OCN-4,5 gradually resembled that of OCN with obvious OCN weight loss steps, and the decomposition was complete at temperatures higher than 700 °C. In short, the CS-OCN graft polymer still possessed good thermal stability.

**Figure 3:**
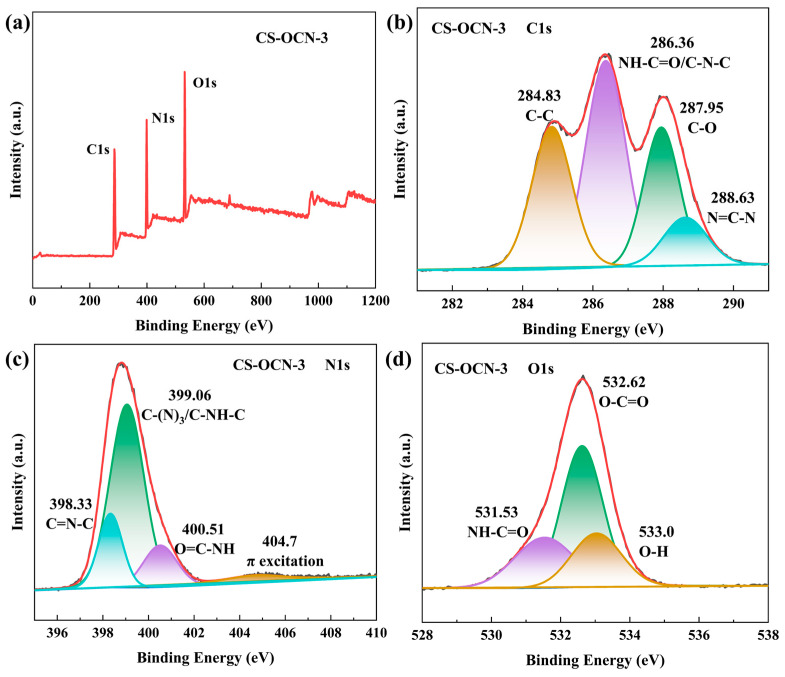
(**a**) XPS Survey, high-resolution (**b**) C 1s, (**c**) N 1s and (**d**) O 1s spectra of CS-OCN-3.

As a typical sample, the chemical composition and chemical state of CS-OCN-3 were investigated by XPS. The sample was mainly composed of C, N and O elements, as shown in Figure 3. The oxygen content in OCN was 12.22%, while it was increased in CS-OCN, which was about 20.45% (Table S2). The increase in O element content may come from CS and the absorption of H_2_O and CO_2_ on the sample surface. The C 1s spectra (Figure 3b) showed a peak at 286.36 eV, which could be attributed to the generation of the amide bond (O=C-NH/C-N-C), and the peak at 287.95 eV corresponded to the C-O bond [51]. The peaks at 288.63 and 284.83 eV can be attributed to N-C=N and C-C, respectively, which were consistent with the structure of the tri-s-triazine unit of the OCN framework [52]. As shown in Figure 3c, the N 1s spectra showed three peaks at 398.33, 399.06 and 400.51 eV, corresponding to C=N-C, N-(C)_3_/C-N-H and O=C-NH, respectively [53]. The O1s spectra (Figure 3d) showed three peaks at 531.53, 532.62 and 533 eV, which belong to the O=C-NH and O-C=O groups and O-H, respectively [54]. Therefore, this evidence of newly generated amino bonds indicated that CS was grafted onto the OCN surface by forming covalent bonds.

### 3.2. Photocatalytic Performances of Prepared Materials

#### 3.2.1. Optical Performance

**Figure 4:**
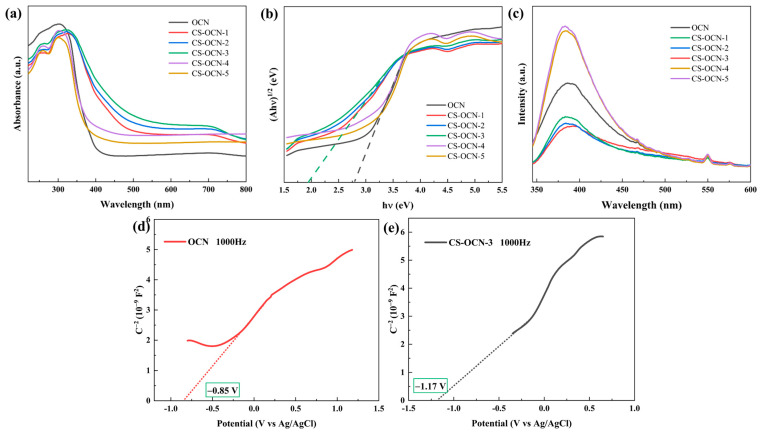
(**a**) UV-Vis light response, (**b**) Band gap energy, (**c**) PL spectra of OCN and CS-OCN, and Mott–Schottky plots of (**d**) OCN and (**e**) CS-OCN-3.

Figure 4a shows the UV-Vis diffuse reflectance spectra of OCN and CS-OCN. There was a strong absorbance at 300–400 nm, and the absorption of porous CS-OCN was red-shifted with the increase in CS content. The maximum redshift from 400 nm of OCN to approximately 600 nm of CS-OCN-3 indicated that the visible light absorption could be realized. The forbidden band width of the semiconductor was calculated by Equation (2).

(2) $${\left(\alpha h\nu \right)}^{\frac{1}{n}}=A\left(h\nu -E_{g}\right)$$

where α is the absorbance index, h is Planck’s constant, ν is the frequency, $E_{g}$ is the semiconductor forbidden band width, A is a constant, OCN is an indirect band gap semiconductor and *n* is taken as 2.

The results are shown in Figure 4b, in which the forbidden band width of OCN was 2.78 eV, while that of CS-OCN-3 became about 2.0 eV. It could be concluded that the appropriate ratio of grafting CS was beneficial to the improvement of visible light utilization, which was expected to realize better photocatalytic activity.

To further investigate the recombination rate of electron–hole pairs generated in the photocatalytic reaction, steady-state photoluminescence (PL) spectroscopy was performed at an excitation wavelength of 325 nm. As shown in Figure 4c, the PL of OCN showed a strong emission concentrated at 350–475 nm, which was the intrinsic fluorescence signal of OCN. The lower PL intensity was obtained in CS-OCN-1,2,3 than OCN, probably because the grafted of CS reduced the density of OCN and provided electron transport paths [55]. Nevertheless, PL emission of CS-OCN-4 and CS-OCN-5 increased when CS ratio decreased further, which was not favorable for photocatalytic reactions, it indicated the increased recombination probability of photogenerated electron–hole pairs. The PL results showed that there was an optimal ratio between CS and OCN.

The Mott–Schottky test was adopted to investigate the energy band positions of OCN and CS-OCN-3. As shown in Figure 4d, e, the positive slopes of OCN and CS-OCN-3 indicated typical features of n-type semiconductors [56], and the flat-band potentials (E_f_) of OCN and CS-OCN-3 were −0.85 and −1.17 V (vs. Ag/AgCl), respectively, which could be converted to −0.65 and −0.97 V (vs. NHE) [57,58]. It was reported that the conduction band potential (E_CB_) of most n-type semiconductors was about 0.1–0.2 V lower than the E_f_ [59], and thus, the conduction band potentials of OCN and CS-OCN-3 were −0.85 and −1.17 V (vs. NHE), respectively. According to the empirical equation E_CB_ = E_VB_ − E_g_ and the E_g_ obtained from the Tauc plots, the valence band potentials (E_VB_) of OCN and CS-OCN-3 were 1.93 V and 0.83V, respectively.

#### 3.2.2. Photocatalytic Decomposition

**Figure 5:**
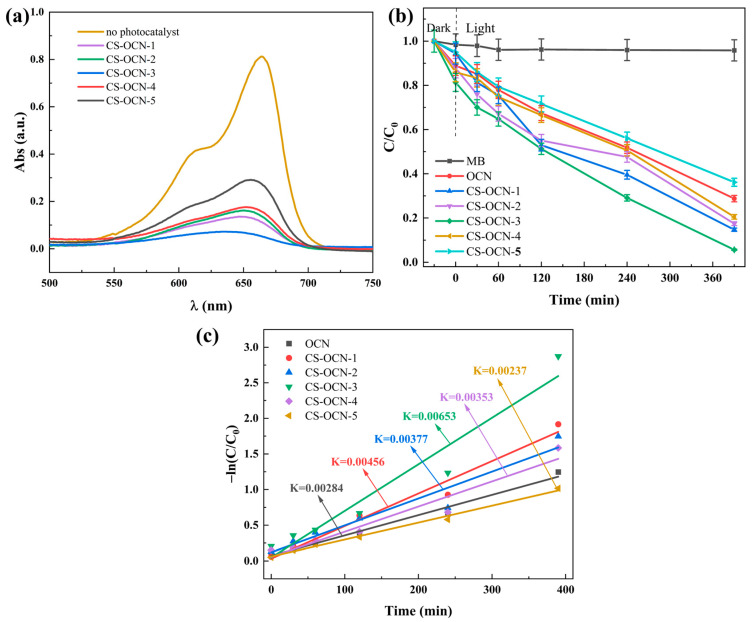
(**a**) UV-Vis absorption spectra, (**b**) Photocatalytic degradation curves and (**c**) Primary kinetic fitting curves of CS-OCN for MB.

To reveal the photocatalytic performance of obtained CS-OCN nanoparticles, MB was applied to evaluate the performance of photocatalytic degradation. Figure 5a showed the UV-Vis absorption spectra of MB photocatalytic degraded by CS-OCN at 390 min reaction time. It is worth noting that there was a significant decrease in the absorbance intensity of MB with CS-OCN compared to blank reference. Among all the tested samples, the absorbance intensity of MB with CS-OCN-3 was the lowest, suggesting that the optimal ratio of CS: OCN was 2:1, and it was the most favorable for degradation performance. The photocatalytic degradation curves of CS-OCN for MB (40 mg/L) are shown in Figure 5b. Before the irradiation, the concentration of MB solution with CS-OCN-3 was already the lowest, which showed that it had the best adsorption for MB. In the blank reference, the concentration of MB solution was almost constant during illumination time, which indicated that the self-degradation of MB solution without photocatalyst under the present measurement conditions was extremely weak. The degradation rates of MB all increased by CS-OCN with different CS contents compared to pure OCN. In particular, the CS-OCN-3 had the best degradation effect, achieving a 94.3% degradation of MB within 390 min. The degradation rate of MB by CS-OCN-5 was the lowest, while it had the highest OCN content. This indicated that the appropriate amount of graft CS could improve the photocatalytic activity of OCN. Meanwhile, the kinetics of MB photocatalytic degradation was investigated. According to the Langmuir–Hinshelwood model, the first-order rate constant (*k*) can be obtained from Equation (3).

(3) $$\ln\left(\frac{C_{t}}{C_{0}}\right)=-kt$$

where $C_{0}$ is the initial concentration of the MB solution, and $C_{t}$ is the concentration at the reaction time *t* under light. As shown in Figure 5c, the *k* value of CS-OCN-3 was about 2.30 times higher than that of pure OCN. The same degradation effect of CS-OCN nanoparticles was observed for 10 mg/L of MB solution (Figure S4). Therefore, it can be assumed that the appropriate amount of graft CS plays an important role in the improvement of OCN photocatalytic performance.

#### 3.2.3. Photocatalytic Antibacterial

**Figure 6:**
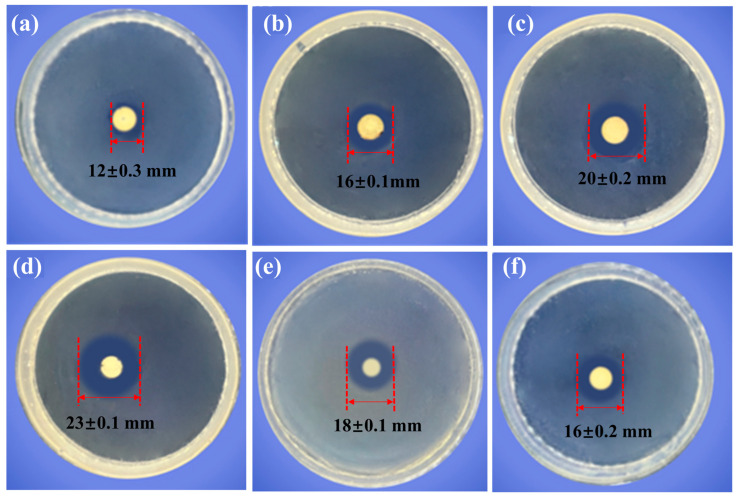
The images of inhibition zones for different samples against *E. coli*. (**a**) OCN, (**b**) CS-OCN-1, (**c**) CS-OCN-2, (**d**) CS-OCN-3, (**e**) CS-OCN-4, (**f**) CS-OCN-5.

**Figure 7:**
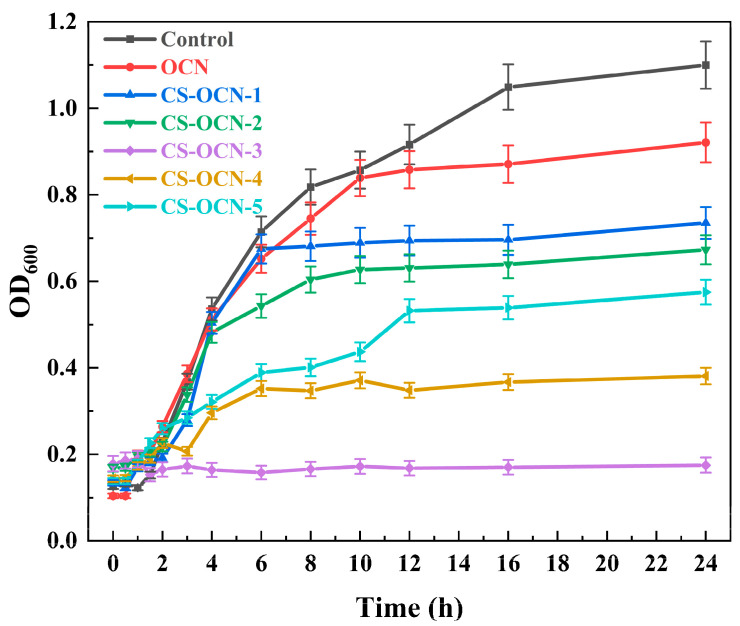
Bacterial growth curves of *E. coli* treated by OCN and CS-OCN.

The antibacterial results of CS-OCN are shown in Figure 6. Compared with pure OCN, the grafting of CS led to an increasing tendency for inhibition circle diameter. In particular, CS-OCN-3 (Figure 6d) had the best bacterial inhibition effect, and the inhibition diameter could reach 23 mm owing to the grafting of CS with OCN. The graft composite may improve electron transport and production of radical, while it enhanced the photocatalytic performance and achieved the photocatalytic synergistic bacterial inhibition effect. In addition, the CS itself may also have an antibacterial effect [60]. However, when the content of OCN was further increased, the inhibition circle diameter became smaller, which indicated the photocatalytic inhibition effect was weakened. Compared with OCN, the CS-OCN showed a significant improvement in the inhibition of *E. coli*. Liu et al. [61] prepared g-C_3_N_4_/CS/PVA films which also showed good antibacterial properties against *E. coli* and *S. aureus*. From the growth curves of *E. coli* in Figure 7, it could be seen that OCN had a certain inhibitory effect on the growth of *E. coli* compared with the control group. The inhibition of *E. coli* growth was enhanced with the increase in CS grafting, among which CS-OCN-3 had the strongest inhibition effect. The antibacterial results were consistent with the results of the inhibition zone measurements, which could further indicate that CS-OCN had excellent visible light antibacterial performance. Shen et al. [62] also found that graphitic carbon nitride-chitosan composites exhibited broad-spectrum biofilm inhibition and eradication against Staphylococcus epidermidis, Pseudomonas aeruginosa PAO1 and Escherichia coli O157: H7 under visible light irradiation.

#### 3.2.4. Determination of ROS

**Figure 8:**
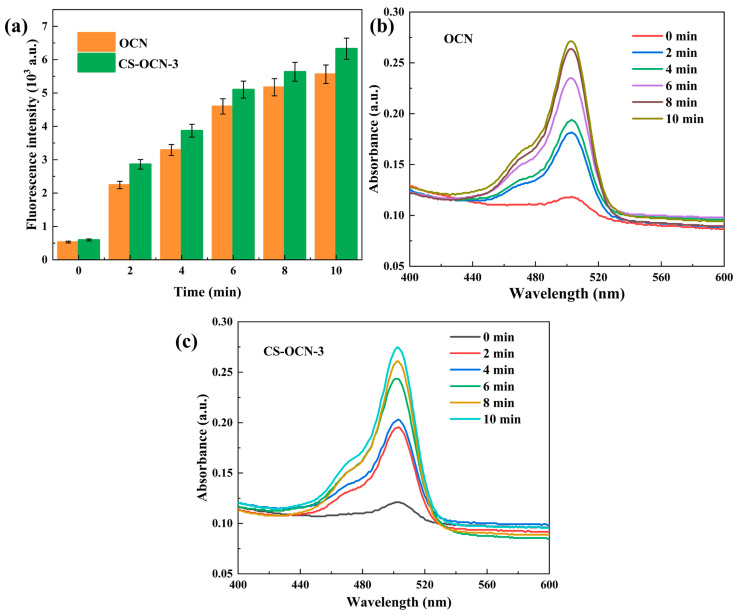
(**a**) Fluorescence intensity of H2DCFDA (λem = 485 nm) with the presence of OCN and CS-OCN-3 with 10 min light irradiation. Absorption spectra of H2DCFDA with the presence of (**b**) OCN and (**c**) CS-OCN-3 with 10 min light irradiation. Light irradiation significantly increased the production of ROS.

It had been reported that irradiation time could affect photocatalytic performance by altering ROS generation [63]. The change in fluorescence intensity in Figure 8a showed that the amount of ROS generated by OCN and CS-OCN-3 gradually increased with the increase in irradiation time to 2, 4, 6, 8 and 10 min. The higher ROS generation of CS-OCN-3 than OCN may be attributed to the facilitative role of CS in the electron transport process, which was beneficial to the generation of more ROS. Similar results were obtained for the absorption spectra of H2DCFDA in the presence of OCN and CS-OCN-3 under 10 min of light irradiation (Figure 8b,c). As the irradiation time increased, the light absorption intensity enhanced, and the ROS production increased. The results showed that the ROS production of CS-OCN-3 was higher than that of OCN, indicating that the grafted CS enhanced the photocatalytic activity.

#### 3.2.5. Photoelectrochemical Studies

**Figure 9:**
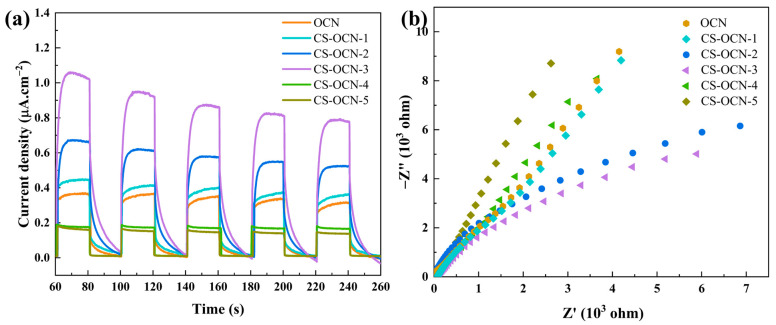
(**a**) Photocurrent responses and (**b**) EIS curves of OCN and CS-OCN.

The transient photocurrent response of the prepared samples was measured to investigate the internal carrier separation transport mechanism (separation and transfer) of OCN and CS-OCN [64]. The light source was turned on and off at 20 s intervals, and the results are shown in Figure 9. From the photocurrent response curves in Figure 9a, it can be seen that the OCN and CS-OCN appeared to respond to the current immediately when the light was turned on, while the current value returned to the initial state when the light was turned off. After five cycles of turning on and off, it was clear that the photocurrent intensity of CS-OCN-3 was the strongest, about 150% more than that of pure OCN. However, the photocurrent intensity of CS-OCN-4,5 became weaker as the OCN content further increased. It showed that the appropriate amount of grafting CS could effectively reduce the electron–hole recombination and provide stable photogenerated electrons for the photocatalytic process [65,66]. In turn, photogenerated electrons increased the reactivity of photocurrents. The results were in general agreement with the UV and PL characterization analysis, thereby improving the reliability of the experiments. The charge carriers transfer resistance of CS-OCN was investigated by EIS Nyquist curves, and the results are shown in Figure 9b. The arc radius of the pure OCN curve was large, which revealed that there was high charge transfer resistance; in other words, the transport of photocatalytic carriers from the interior to the surface was weak [67]. The arc radius of the CS-grafted samples decreased compared with that of the OCN, where CS-OCN-3 was the smallest, which suggested that the appropriate amount of grafting CS contributed to the optimal charge transport capacity [68]. The results above indicated that the optimal ratio of CS and OCN in the work contributed to an optimum value on the properties of charge transport.

## 4. Conclusions

To sum up, CS-OCN nanoparticles were successfully prepared for the first time. The graft ratio of OCN and CS had a significant effect on the efficiency of photocatalytic degradation of MB and the inhibition effect against *E. coli*. At the optimal CS content, the prepared CS-OCN-3 sample had the best photocatalytic degradation efficiency and bacterial inhibition, with 94.3% degradation of MB and a maximum inhibition diameter of 23 mm. Meanwhile, the photocurrent response intensity was about 150% more than that of pure OCN. The best inhibition effect against *E. coli* was also achieved. The enhancement of these results could be due to the synergistic inhibition effect of CS and OCN, and CS may provide paths in electron transport for more ROS. This work provided a rational design for the future intelligent design and preparation of highly efficient OCN-based eco-friendly photocatalysts for the treatment of water pollution.

## Data Availability

Data are available on request from the corresponding author.

## Supplementary Materials

The following supporting information can be downloaded at: https://www.mdpi.com/article/10.3390/polym15071688/s1, Figure S1: SEM images of (a) OCN and (b) CS-OCN-3.; Figure S2: 1 mg/mL OCN and CS-OCN nanoparticles dispersing in PBS solution (a) before and (b) after 3 days.; Figure S3: TGA curves of OCN and CS-OCN.; Figure S4: (a) Photocatalytic degradation curves and (b) Primary kinetic fitting curves of CS-OCN for MB.; Table S1: CrI values for OCN and CS-OCN-3 XRD peaks.; Table S2: The elemental contents of OCN and CS-OCN-3.; Table S3: Comparison of g-C_3_N_4_-based catalysts for dye photodegradation. References [10,32,33,38,69,70,71] have been cited in the Supplementary Materials.
