# Supplementary material for: Chitosan-Grafted Carbon Oxynitride Nanoparticles: Investigation of Photocatalytic Degradation and Antibacterial Activity
Source: Polymers (Basel). 2023 Mar 28;15(7):1688. doi: 10.3390/polym15071688 (PMC10096967; doi:10.3390/polym15071688)
Supplement: Supplementary file 1 [file polymers-15-01688-s001.zip › polymers-2252771-supplementary.pdf]

Table S1. CrI values for OCN and CS-OCN-3 XRD peaks.

**Samples**	**CrI (%) at 2θ**		
	**12.2**	**19.9**	**27.4**
**CN**	54.8	0	86.4
**OCN**	13.6	0	82.9
**CS-OCN-2**	46.3	71.9	33.6
**CS-OCN-3**	74.6	72.4	59.5
**CS-OCN-5**	80.9	30.4	88.2

Table S2. The elemental contents of OCN and CS-OCN-3.

**Name**	**OCN**	**CS-OCN-3**
**C1s**	38.77%	52.89%
**N1s**	49.01%	26.66%
**O1s**	12.22%	20.45%

Table S3. Comparison of g-C_3N_4-based catalysts for dye photodegradation

Photocatalyst Type (Mass to Solution VolumeRatio)	Dye concentration	Light Source	Degradation efficiency	Reference
AgCl/CNTs/g-C_3N_4 (50 mg to 100 mL)	20 mg/L	250 W Xe lamp	86.44% (90 min)	[1]
pCN/TNs	20 mg/L	500 W Xe lamp	86.2% (120 min)	[2]
PoPD/AgCl/g-C_3N_4(50 mg to 100 mL)	20 mg/L	250W Xe lamp	83.06% (120 min)	[3]
OCN-24-550(50 mg to 50 mL)	20 mg/L	300 W Xe lamp (λ>420 nm)	85.76% (120min)	[4]
Mo_2C/g-C_3N_4 type-I Heterojunction ((30 mg to 30 mL)	20 mg/L	300 W Xe lamp (λ > 420 nm)	91.6% (60 min)	[5]
CS/g-C_3N_4 doped $Ba(OH)_2$(10 mg to 40 mL)	10 mg/L	visible light (400–800 nm)	89.39% (120 min)	[6]
CQD-C_3N_4	10 mg/L	250 W Xe lamp (λ>420 nm)	78.6% (240 min)	[7]
CS-OCN-3((30 mg to 30 mL)	40 mg/L	200 W Xe lamp (λ>420 nm)	94.3% (390 min)	This work

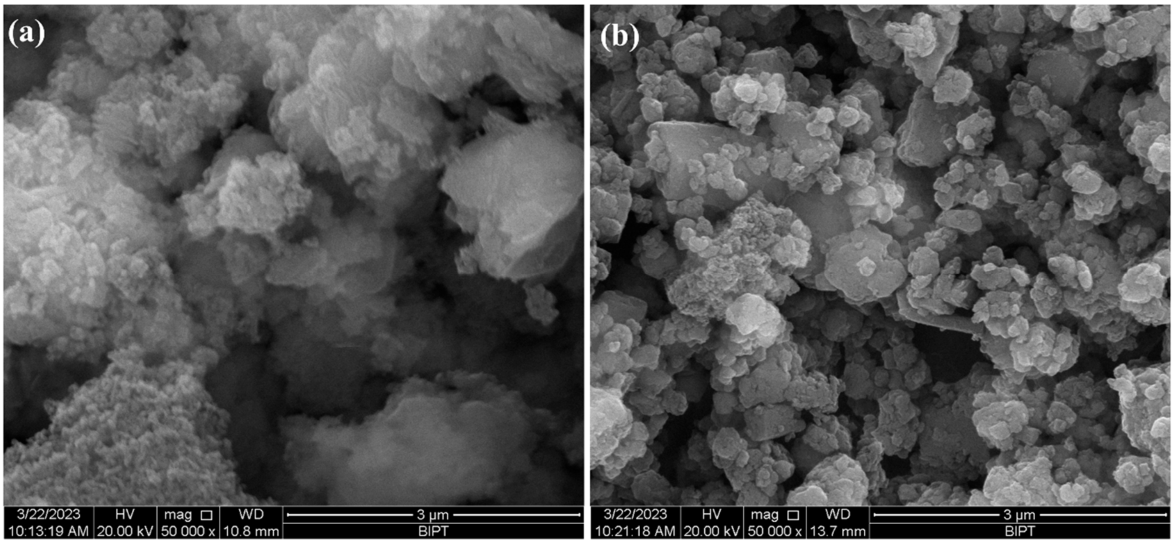

Figure S1. SEM images of (a) OCN and (b) CS-OCN-3.

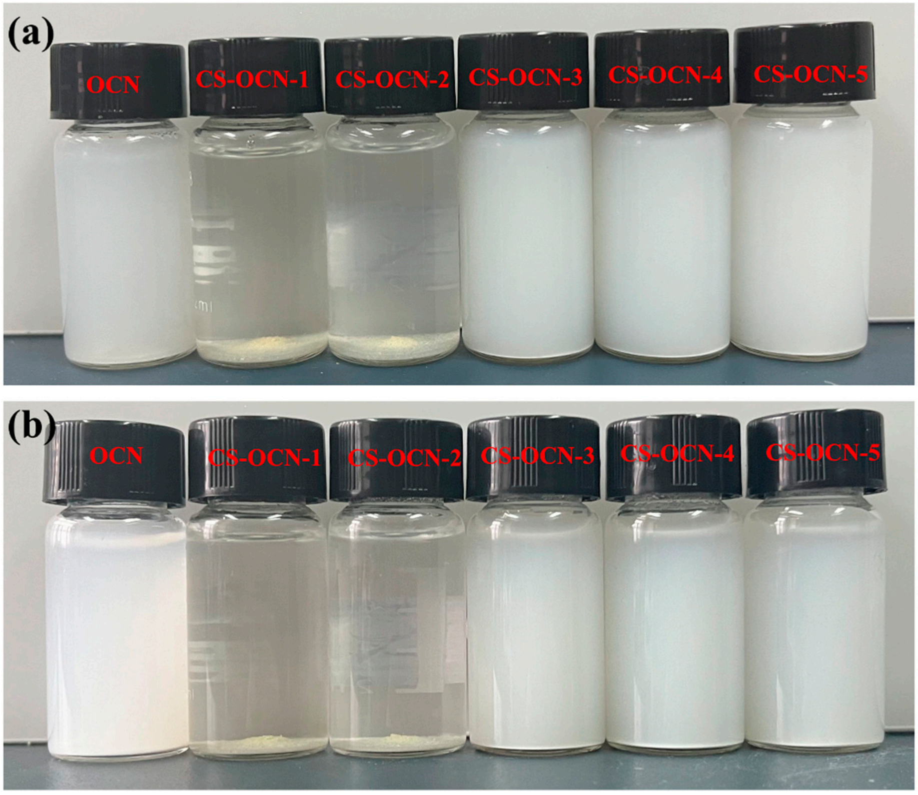

Figure S2.1 mg/mL OCN and CS-OCN nanoparticles dispersing in PBS solution (a) before and (b) after 3 days

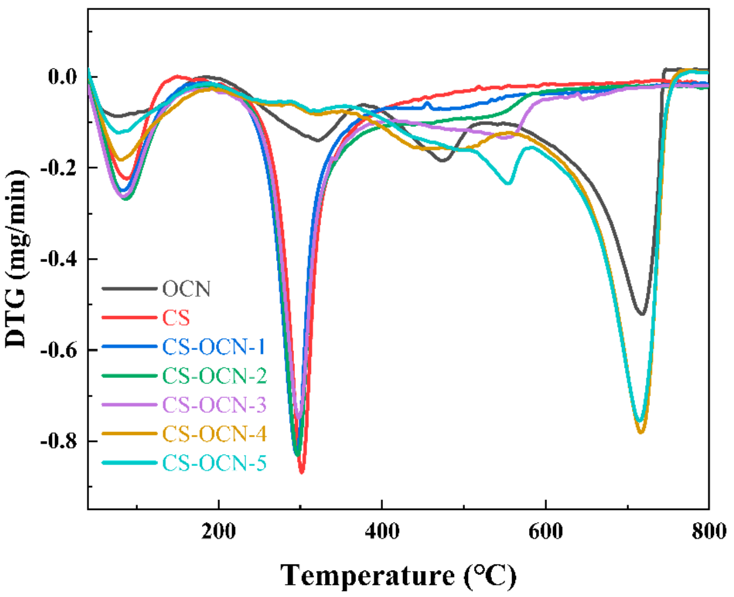

Figure S3. TGA curves of OCN and CS-OCN.

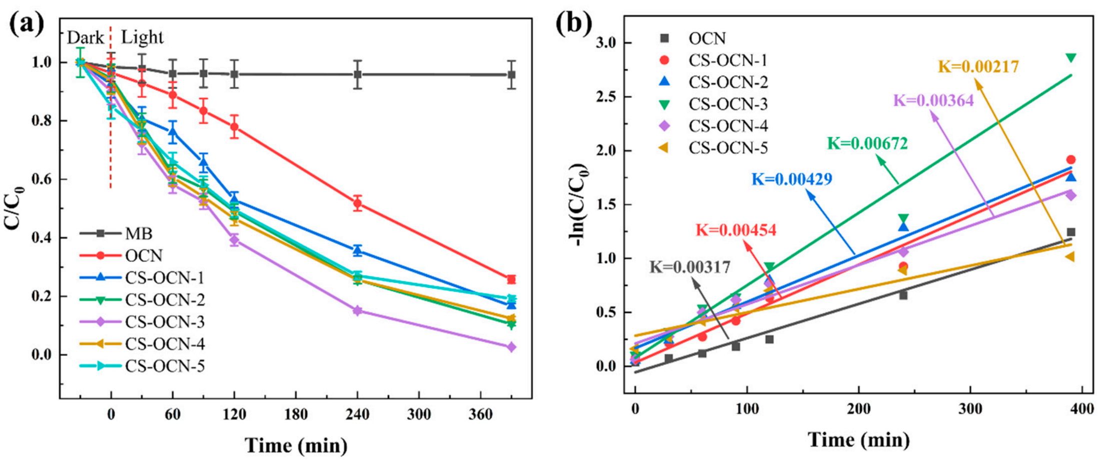

Figure S4. (a) Photocatalytic degradation curves and (b) Primary kinetic fitting curves of CS-OCN for MB

[1] Liu, C.; Tang, Y.; Huo, P.; Chen, F. Novel AgCl/CNTs/g-C_3N_4 nanocomposite with high photocatalytic and antibacterial activity, Mater. Lett. 2019, 257, 126708.

[2] Liu, X.; Yang, Z.; Yang, Y.; Li, H. Construction of carbon quantum dots sensitized porous carbon nitride/titanium dioxide nanosheets for enhancing visible light photocatalytic degradation of tetracycline, J. Environ. Chem. Eng. 2022, 10, 108083.

[3] Sun, L.; Liu, C.; Li, J.; Zhou, Y.; Wang, H.; Huo, P.; Ma, C.; Yan, Y. Fast electron transfer and enhanced visible light photo-catalytic activity by using poly-o-phenylenediamine modified AgCl/g-C_3N_4 nanosheets, Chin. J. Catal. 2019, 40, 80-94.

[4] Guo, H.; Niu, C. G.; Feng, C. Y.; Liang, C.; Zhang, L.; Wen, X. J.; Yang, Y.; Liu, H-Y.; Li, L.; Lin, L. S. Steering exciton dissociation and charge migration in green synthetic oxygen-substituted

ultrathin porous graphitic carbon nitride for boosted photocatalytic reactive oxygen species generation. Chem. Eng. J. 2020, 385, 123919.
[5] Zhang, C.; Zhou, Y.; Wang, W.; Yang, Y.; Zhou, C.; Wang, L.; Lei, L.; He, D.; Luo, H.; Huang, D. Formation of Mo_2C/hollow tubular g-C_3N_4 hybrids with favorable charge transfer channels for excellent visible-light-photocatalytic performance. Appl. Surf. Sci. 2020, 527, 146757.
[6] Ikram, M.; Haider, A.; Naz, S.; Bari, M. A.; Haider, J.; Ul-Hamid, A.; Nabgan, Walid.; Imran, Muhammad.; Nazir, Ghazanfar.; Ali, S. Chitosan and carbon nitride doped barium hydroxide nanoparticles served as dye degrader and bactericidal potential: A molecular docking study. Int. J. Biol. Macromol. 2023, 224, 938-949.
[7] Hong, Y.; Meng, Y.; Zhang, G.; Yin, B.; Zhao, Y.; Shi, W.; Li, C. Facile fabrication of stable metal-free CQDs/g-C_3N_4 heterojunctions with efficiently enhanced visible-light photocatalytic activity. Sep. Purifi. Technol. 2016, 171, 229-237.
